# Subword Representations Successfully Decode Brain Responses to Morphologically Complex Written Words

**DOI:** 10.1162/nol_a_00149

**Published:** 2024-09-11

**Authors:** Tero Hakala, Tiina Lindh-Knuutila, Annika Hultén, Minna Lehtonen, Riitta Salmelin

**Affiliations:** Department of Neuroscience and Biomedical Engineering, Aalto University, Espoo, Finland; Aalto NeuroImaging, Aalto University, Espoo, Finland; Department of Psychology and Speech-Language Pathology, University of Turku, Turku, Finland; Centre for Multilingualism in Society Across the Lifespan, University of Oslo, Oslo, Norway

**Keywords:** decoding, MEG, multimorphemic words, statistical morphemes, word2vec

## Abstract

This study extends the idea of decoding word-evoked brain activations using a corpus-semantic vector space to multimorphemic words in the agglutinative Finnish language. The corpus-semantic models are trained on word segments, and decoding is carried out with word vectors that are composed of these segments. We tested several alternative vector-space models using different segmentations: no segmentation (whole word), linguistic morphemes, statistical morphemes, random segmentation, and character-level 1-, 2- and 3-grams, and paired them with recorded MEG responses to multimorphemic words in a visual word recognition task. For all variants, the decoding accuracy exceeded the standard word-label permutation-based significance thresholds at 350–500 ms after stimulus onset. However, the critical segment-label permutation test revealed that only those segmentations that were morphologically aware reached significance in the brain decoding task. The results suggest that both whole-word forms and morphemes are represented in the brain and show that neural decoding using corpus-semantic word representations derived from compositional subword segments is applicable also for multimorphemic word forms. This is especially relevant for languages with complex morphology, because a large proportion of word forms are rare and it can be difficult to find statistically reliable surface representations for them in any large corpus.

## INTRODUCTION

Corpus-semantic vector spaces are a useful approach to quantify representations of words and their parts, and their semantic relationships. In these models, words are expressed as vectors in a space which represents a fuzzy continuum of semantic, syntactic, and functional properties, based on ideas of Harris (1954) and Firth (1968). It has been shown that these word vectors can be correlated with portions of neural activity (Mitchell et al., 2008; Xu et al., 2016). In the present study, we extend this idea of decoding word-evoked brain activation using a corpus-semantic vector space to examine whether multimorphemic words can be represented compositionally as a sum of their constituent units and whether there are limits to building such compositions. Specifically, in addition to constructing a vector space for words, we train alternative vector space models with various alternative word segmentations. The compositional word representations are especially important in languages with a high number of multimorphemic words. We conduct our study using Finnish, a language with rich inflectional morphology. The language is agglutinative, that is, the morphemes are concatenated when embedded in a complex word. Therefore, it should be possible to represent complex Finnish words compositionally as a simple sum of distinct morphemes.

Word decoding studies seek to determine a generalized function that maps the corpus-semantic vector space populated by words to brain activity recorded during word processing. Successful decoding seems to imply some level of correspondence between the corpus-semantic model and the brain activity. In recent years, this approach has demonstrated success: For example, Djokic et al. (2020) provide evidence that compositional models effectively capture patterns of human meaning representation in the processing of both literal and metaphoric language usage. For a recent review of studies addressing the neural decoding of semantic concepts, see Rybář and Daly (2022). Using this methodology, it has been possible, for example, to propose a thematic distribution of word representations in the brain (Hultén et al., 2021; Huth et al., 2016) and to demonstrate that presenting only partial information about an object suffices to evoke its complete semantic representation (Kivisaari et al., 2019). Although not many word decoding experiments have explicitly focused on subword properties, some studies, such as Huth et al. (2016), included stimuli with inflected words (e.g., those ending in -ing or -ed).

A popular method for constructing semantic spaces for word decoding studies is word2vec (Mikolov, Chen, et al., 2013; Mikolov, Sutskever, et al., 2013). In word2vec, the vectors are trained by analyzing the context in which a word appears, typically considering a specific number of words before and after the target word. The method enables interesting arithmetic operations on the vectors, such as the famous example king − man + woman = queen. This suggests a technique for building vectors for longer words or unseen words in training, by deconstructing them into smaller components, such as syllables or subword units, and then combining their respective vectors.

In linguistics, the smallest meaningful unit of language is the morpheme (Anderson, 2019). A complex word, such as “un + talk + able, ” is composed of multiple morphemes that each carry distinct semantic information. If a word is not recognized as a whole, the perceiver can determine the meaning by analyzing the morphemes (Diependaele et al., 2012). Besides linguistics, modeling morphology is an important problem in natural language processing (NLP) applications. For example, in speech recognition applications it is often necessary to reduce lexicon sizes in highly inflected languages. There has been success in applying information-theoretical principles to automate morphological analysis without recourse to linguistic rules. Here, as an example of models used in language technology applications, we look into Morfessor, which generates statistically motivated word pieces that often resemble linguistic morphemes (Creutz & Lagus, 2007; Virpioja et al., 2013). It aims to segment words in such a way that the total set of word pieces would optimally describe the training corpus. Morfessor has been successfully used to provide quantitative predictions and insights for reaction times, eye-tracking, and brain activity measures during visual word recognition tasks (Hakala et al., 2018; Lehtonen et al., 2019; Virpioja et al., 2011; Virpioja et al., 2018). In the present study, to address various potential subword representations, we construct and evaluate multiple distinct models. Two of these models are specifically designed to capture Finnish morphology: The first model employs linguistic analysis for word segmentation, while the second uses the Morfessor model. Additionally, we analyze segmentation models that are not sensitive to morphology. These include 1-gram, 2-gram, and 3-gram models, where each word is segmented into 1, 2, or 3 character segments, respectively. We also employ a model that segments words randomly and a whole-word model with no segmentation. Corpus-derived vector representations for the word labels and individual segment labels are constructed using the word2vec embedding method.

For neurocognitive validation of these various corpus-based models, we use magnetoencephalography (MEG) data collected during visual word recognition, known to represent a sequence of distinct neurofunctional responses (Salmelin, 2007). After presentation of a single word, the first salient response in the occipital cortex at around 100 ms from the word onset is modulated by low-level visual complexity (Tarkiainen et al., 1999). The following occipitotemporal activation at 150–200 ms shows increased activation to alphabetic input compared to symbols (Parviainen et al., 2006; Tarkiainen et al., 1999). Activation in this time window has also been associated with visual word forms and proposed to index early-stage morphemic segmentation as the activation seems to be modulated by the transition probability between the word stem and suffix (Lewis et al., 2011). Subsequent sustained activation in temporal cortices, with left-hemispheric predominance, reaches the maximum at around 400 ms after the word onset. This response is modulated by semantic congruence of a word in the context, with more unlikely words associated with stronger response (Halgren et al., 2002; Helenius et al., 1998; Service et al., 2007). However, the exact properties of the response are complex and depend on the particular circumstances and task demands (Kutas & Federmeier, 2011). Based on previous work (Chan et al., 2011; Hultén et al., 2021; Simanova et al., 2010; Sudre et al., 2012; Xu et al., 2016), we expect reasonable decoding performance using the whole-word model within the time window of the sustained response, from approximately 200 ms to 600 ms. We investigate whether subword models will yield successful decoding of brain responses, similar to the whole-word model, and whether linguistically or statistically motivated subword segmentation models result in better decoding accuracy than character-based or random segmentation.

## MATERIALS AND METHODS

### Participants

We analyzed data from 20 participants, native Finnish speakers, all of whom were right-handed as per the Edinburgh Handedness Inventory (Oldfield, 1971) and reported no neurological problems. The age range was 20–37 years (mean 24.4, *SD* 6.4), and 11 participants were female. Data from three additional participants were collected but discarded as the percentage of artifact-free trials with correct responses was less than 85%. The study was approved by the ethics committee of the Hospital District of Helsinki and Uusimaa. All subjects of the study have given their informed consent. Brain activation during the experiment was recorded with a Vectorview MEG system (Elekta Ltd, Helsinki, Finland), at the MEG Core, Aalto NeuroImaging.

### Stimuli

**Figure 1.**
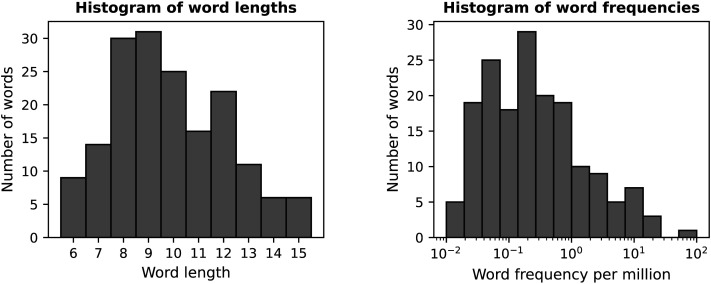
Descriptive statistics of the word set used in the study. Left panel: Distribution of word lengths in characters. Right panel: Frequencies of words per million words.

The words analysed in this study consisted of 170 multimorphemic Finnish words that were randomly selected nouns from the Morpho Challenge 2007 corpus (Kurimo et al., 2008), comprising around 55 million word tokens of which 2.2 million are unique. For this set of 170 words, the word length varied from 6 to 15 characters (mean 9.9, *SD* 2.3) and the word frequency, calculated using the Finnish internet corpus (Luotolahti et al., 2015), varied from 0.02 to 60 words per million. The number of linguistically defined morphemes varied between two and five. Histograms of word lengths and frequencies are shown in Figure 1.

These words were linguistically multimorphemic according to a commercial language structure analysis tool (Lingsoft Oy, Turku, Finland), that is, consisting of root lemma and at least one inflectional or derivational affix. Manual inspection confirmed they were indeed multimorphemic. Focusing only on the multimorphemic words among the stimuli ensured that a categorical division between mono- and multimorphemic words could not drive the decoding performance. In addition, a corpus frequency of at least 50 instances was required, in order to construct reasonable semantic models for whole word forms with the word2vec algorithm (see Brain Decoding).

We reuse brain signals evoked by these 170 multimorphemic words in a published word recognition study (Hakala et al., 2018). In that experiment, the stimuli consisted of 480 Finnish words (half of them monomorphemic, the other half multimorphemic), 360 pseudowords, and additional nonword stimuli, which were included for functional localization of specific word-reading related responses, employed in that study.

### Procedure

During the MEG recording, the participant was seated in a magnetically shielded room, their head inside a Vectorview MEG system (Elekta Ltd, Helsinki Finland). The MEG system contains, at 102 recording sites, 204 planar gradiometers (2 orthogonally oriented coils per site) and 102 magnetometers. The head position in the MEG helmet was measured using indicator coils attached to the scalp. Four electrodes attached next to the eyes were used to record blinks and eye movements (electrooculogram, or EOG). The stimulus items were individually projected onto a screen situated 140 cm from the participant’s forehead. The stimuli were presented in black monospace Courier New font against a gray background, with a visual angle ranging from 2.5 to 6.2 degrees, depending on the length of the item. Trials started with a fixation cross that was displayed for 500 ms. Thereafter, the stimulus was displayed for 1,500 ms. A new trial started immediately after that. The participant was instructed to indicate whether the displayed item was a real Finnish word or not. The *yes*/*no* answer was given by lifting the right or left index finger (balanced across participants). If the correct answer was not given within 1,500 ms from the stimulus onset, the trial was discarded from further analysis. The order of the stimuli was randomized, and the experiment was divided into six blocks, each lasting for around 10 min, with a short resting break between the blocks.

### MEG Data Preprocessing

The MEG data were online band-pass filtered at 0.03–200 Hz and sampled at 1000 Hz. The continuously recorded raw data were first cleaned from external artifacts with the spatiotemporal signal space separation method tSSS (Taulu & Simola, 2006), implemented in MaxFilter software (Elekta Oy), and then low-pass filtered at 40 Hz using Hamming-windowed zero-phase FIR filter with automatic selection of length, implemented in the MNE toolbox (Version 0.19.0; Gramfort et al., 2013). The head positions of each participant were computationally aligned into a common position with respect to the MEG helmet using the MaxFilter software. Data inspection confirmed that online high-pass filtering and tSSS effectively mitigated any low frequency drifts and no additional offline high-pass filtering was performed.

Electromagnetic artifact signals due to blinks and eye movements were removed using independent component analysis. Components with high correlation with EOG channels and spatial topography typical of ocular artifacts were manually identified and removed (1–3 components per participant), and the MEG signal was subsequently reconstructed using the MNE toolbox.

The MEG data analysis was done using the planar gradiometers. For decoding of presented words using MEG data, gradiometers have been shown to perform better than magnetometers (Dash et al., 2021). The data were epoched using a time window spanning from −200 ms to 800 ms with respect to the stimulus onset, and baseline corrected by subtracting the mean amplitude of the 200-ms pre-stimulus time window. Epochs with gradiometer values exceeding 3,000 fT/cm were discarded. Epochs that preceded an incorrect or missing response were discarded.

In the original word recognition experiment, each item was shown only once per participant in order to avoid priming effects. As the signal-to-noise ratio is low for single trials, MEG responses for each individual item were obtained by averaging the single trials of that item across participants at the sensor level. Averaging source activity over participants was successfully used in the previous study on these data (Hakala et al., 2018). In the present study, sensor-level data were used, as decomposing the signal into source estimates would only distribute the sensor-level information to a less condensed form, which increases the degrees of freedom and tends to weaken the decoding result (Sato et al., 2018).

The mean number of discarded trials per stimulus word was 2.7 with a standard deviation of 2.4. If data from more than three participants for a given word had to be discarded, that word was not selected for use in the present study. Thus, in the following decoding phase, the MEG response for each of the 170 words was the average over at least 17 participants.

### Brain Decoding

**Figure 2.**
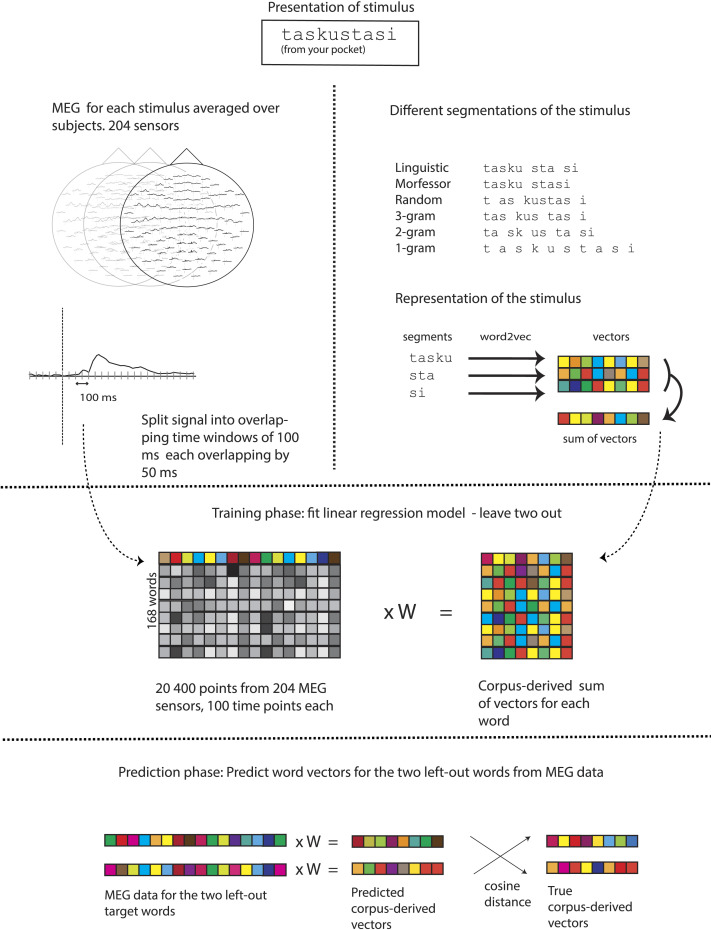
The experiment and analysis workflow. Top left: Magnetoencephalography (MEG) data to each stimulus word are recorded during a lexical decision task. Top right: All words in the training corpus are segmented into subword segments using one of the segmentation schemes. Vector representations for individual segments are constructed using the word2vec skip-gram algorithm. Vectors for each stimulus word are constructed by summing the subword vectors of that word. Middle: The optimal linear mapping between MEG data and word vectors is trained. Bottom: Words that were not part of the training are used to assess the accuracy of the learned mapping.

The schematic of the analysis is shown in Figure 2. The idea of neural decoding is to find the optimal correspondence between the vector space X representing the measured brain activity and corpus-semantic vector space Y of the model. That is, we are looking for the best linear approximation for the function *f*: *X* → *Y*.

For the decoding, we used ridge regression, which is a multivariate linear regression with L2 regularization (Palatucci et al., 2009). The model creates a linear mapping between the input matrix X and target matrix Y. The columns of both the input and target matrices were *z*-transformed before entering them into the linear regression. L2 regularization assigns a penalty to the sum of the squared magnitudes of the coefficients (i.e., the L2 norm of the coefficients), ensuring that none of them excessively dominates the regression model. This regularization approach effectively mitigates the problem of overfitting, particularly in the context of high-dimensional data, and helps to stabilize the numerical solution. We applied the RidgeCV function from the scikit-learn library for our analysis (Pedregosa et al., 2011). The regularization parameter (alpha) was automatically tuned by iterating over logarithmically spaced range of alpha values (from 10^−5^ to 10^5^ over 100 points). A unique alpha was optimized for each target dimension. This parameter search was validated using leave-two-out cross-validation. The model was trained independently for consecutive 100-ms time windows of MEG data that overlapped by 50 ms. Each time window contained 100 time points, corresponding to the 1000 Hz sampling rate.

The decoding accuracy was evaluated by the two versus two test, that is, the training step was performed by omitting two words, which were then used to test the classification accuracy, and the procedure was repeated for all combinations of word pairs, similarly to Mitchell et al. (2008). Successful classification means that when the test words *w*_1_ and *w*_2_ are projected from the measurement space to the corpus-semantic vector space, the sum of the distances from their projected positions (*p*_1_, *p*_2_) to their actual positions (*a*_1_, *a*_2_) using the cosine metric is smaller than the sum of the cross distances (*p*_1_ to *a*_2_ and *p*_2_ to *a*_1_), that is, *d*(*p*_1_, *a*_1_) + *d*(*p*_2_, *a*_2_) < *d*(*p*_1_, *a*_2_) + *d*(*p*_2_, *a*_1_). The statistical significance of the overall decoding accuracy was estimated using 1,000 permutations by randomizing the (whole) word labels. The word-label significance threshold was set at the 95th percentile of the distribution obtained from these permutations.

When word vectors are constructed by summing the vectors of subword segments, it is likely that words containing identical segments cluster together in the word vector space to a certain extent, regardless of the nature of the individual segment vectors. This happens because when vectors are composed of component vectors, any shared components tend to align the vectors in a similar direction. The resulting model may then represent word similarities that are a byproduct of this summing process. This may enable successful decoding even when the individual segment vectors lack useful information, as the test words share segments with the words in the training set. To evaluate the significance of semantic information in the segment vectors, we further conducted a segment-based permutation test. In this test, rather than permuting word labels, we permuted the segment labels as follows.

**Figure 3.**
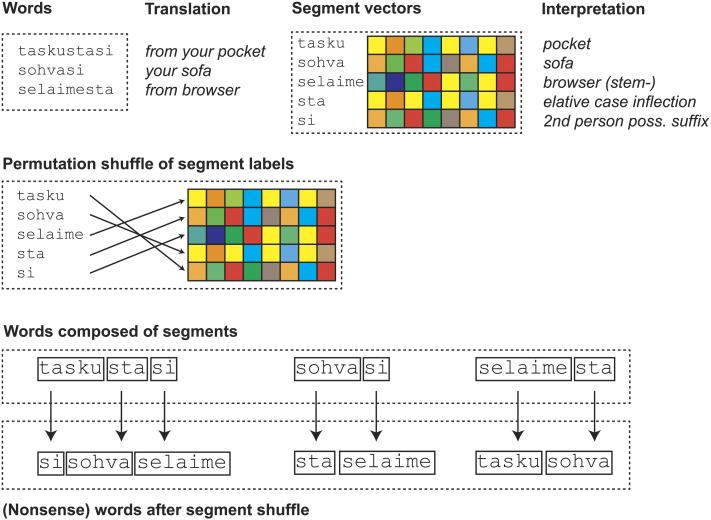
Illustration of the segment-label permutation procedure with a set of three words and linguistic segmentation. Top: The set of words and the set of segment vectors. Middle: The pairing of segment labels with segment vectors is shuffled. Bottom: Words are composed of segments and represented by the sums of corresponding segment vectors. Shuffling the segment labels results in a set of word vectors that represent nonsensical words, effectively scrambling the semantic information. The procedure retains the inherent structure of the word vector set, which arises due to patterns of shared segments within the word set. If two words contain a common segment, the corresponding nonsensical words will also share a segment.

Consider a set of segments comprising all word segments from the set of words under study. Each segment label is associated with a unique segment vector. We shuffled the pairing between segment labels and segment vectors so that each segment label became uniquely associated with a randomly selected segment vector from the set. Subsequently, we constructed word vectors as previously, but utilizing this permuted set of segment vectors. The procedure is illustrated in Figure 3.

The overall accuracy in the decoding task was then calculated, and the process was repeated 1,000 times, each time reshuffling the pairing between segment labels and segment vectors. The segment-label permutation threshold was set at the 95th percentile of the obtained distribution. The significance of the decoding accuracy with the original ordering was then assessed against this threshold. Additionally, by comparing the significance thresholds obtained from segment-label permutation with those from word-label permutation, we can gain insight into whether the semantic model contributes to successful decoding. If the significance threshold for segment-label permutation test is substantially higher than that for word-label permutation, it suggests that decoding is possible regardless of the identity of segment vectors that make up the word vectors. In this case, the decoding works because the word vectors align due to the common segment vectors. If, however, the threshold for segment-label permutation is similar to that of word-label permutation, this suggests that replacing segments of a word is comparable to changing the entire word, and the success of decoding depends on the information contained in the subword segment vectors that is provided by the corpus-semantic model.

### Word Segmentations

The training corpus used in the study was the Finnish internet corpus consisting of a total of 3.6 billion words (Luotolahti et al., 2015). The whole-word model was trained directly for the surface word forms as they appeared in the corpus. For the subword-based models, the words in the corpus were segmented into morphemic units before training the corpus-semantic models. The segmentation to linguistic morphemes was done using a commercial linguistic analysis software for Finnish by Lingsoft Oy (Turku, Finland). The analyzer uses hand-crafted rules that produce good, although not perfect, linguistically defined segmentation. The resulting corpus contained 5.4 × 10^9^ morphemes, of which 9 × 10^5^ were unique. The number of linguistic morphemes per target word varied between two and five.

The domain of NLP offers means for statistical morpheme segmentation. As an example of such a model, we use the Morfessor model (Creutz & Lagus, 2007; Virpioja et al., 2013), in which words are assumed to be composed by concatenation of morphemic units, for example, think + er. The cost of a word is then calculated by summing the cost of individual morphemes, e.g., I(thinker) = I(think) + I(er), where the cost I is the surprisal or negative log probability of the word segment. The morphemes are not defined a priori; instead, they are learned from data during the model training in an unsupervised manner. Morfessor seeks to determine a set of morphemic units that minimize the average surprisal of all words in the corpus, while trying to keep the set of morphemes as small as possible following the minimum description length principle (Rissanen, 1978). The morphemic units that emerge from the Morfessor model approximate linguistic morphemes but are generally somewhat longer, and words with a high-frequency surface form are usually left unsegmented (Virpioja et al., 2018). The number of morphemes per target word, determined by Morfessor, varied between 1 and 3 (*SD* 0.46). Deviation from linguistic standard can be an undesired property if the task is to find linguistic morphemes, but it resonates well with both the idea of neural optimization (Hopfield & Tank, 1985) and psycholinguistic models that consider the balance between the cost of storing words as explicit representations and the additional computational cost that may be required for segmentation and combination of distinct subword segments (Kuperman et al., 2009; Lehtonen et al., 2006). It may also reflect aspects of the brain’s processing, particularly if the brain similarly avoids decomposing some high-frequency inflected words.

Several recent studies have shown that statistically derived units indeed offer a plausible description of how humans might process morphology. Results from word recognition studies that have recorded reaction times (Virpioja et al., 2011; Virpioja et al., 2018), eye movements (Lehtonen et al., 2019), and MEG (Hakala et al., 2018) have shown that the quantitative word surprisal values derived from the Morfessor model were associated with longer reaction times, longer fixations, and increased amplitude of evoked activity at the bilateral middle superior temporal cortices. Furthermore, these associations were stronger and partially independent from those obtained for common psycholinguistic variables, including frequency measures, which have typically proven the strongest predictors of reaction times (Brysbaert et al., 2016).

Morfessor was trained on the Morpho challenge 2007 corpus (Kurimo et al., 2008; Virpioja et al., 2013). The morphological segmentation of the Finnish internet corpus resulted in 5.04 × 10^9^ segments. Of these, 1.2 × 10^5^ were unique. Thus, both the linguistic and statistical morphological models segmented words into an approximately equal number of parts, but the lexicon in the statistical model was notably smaller. The segmentations for each word used in this experiment are provided in the Supporting Information, available at https://doi.org/10.1162/nol_a_00149. Of the 170 words used in the experiment, 58 words were segmented identically by the two morphological analyzers, and in 71 cases the segmentation by Morfessor was incomplete or completely unsegmented (i.e., two or more segments were joined together) compared to the linguistic segmentation. Details for different types of segmentation differences are given in Table 1.

**Table 1.** Performance of the statistical Morfessor method compared against the linguistic segmentation

**Category**	**Number of words**
Identical segmentation	58
Incomplete segmentation	40
Unsegmented	31
Incorrect segmentation, stem	28
Incorrect segmentation, suffix	13
Total	170

In addition to the morphology-based segmentation models, we constructed three character-level n-gram models that segment each word into segments of 1, 2, or 3 characters in length. We also constructed a model that employs random segmentation. The segmentation into random units was done by splitting each word into *n* segments at randomly selected positions where *n* is a random number from the uniform distribution *U*(2, *l*_*w*_*i*__/2) where *l*_*w*_*i*__ is the length of the word *w*_*i*_ being processed. For repeated instances of a particular word in the corpus, identical segmentation was used. We initially tested four separate random segmentation models, each with different random seeds. They all showed similar performance, hence we report here the results of one segmentation.

### Corpus-Semantic Models

In the vector space model, a word is mapped to the vector space as a function of the context in which the word is typically used in the language. For a thorough review on different methodologies for building vector space models, see, e.g., Lenci (2018). Here, the corpus-derived semantic spaces were generated using the word2vec skip-gram algorithm (Mikolov, Chen, et al., 2013). The skip-gram algorithm works by training a neural network with a single hidden layer. The model is usually described and trained for whole words, but here we apply it also to pre-segmented text and therefore refer to segments. Given a segment, the network is trained to predict surrounding segments in some text context. The input layer of the network represents segments as one-hot vectors while the output layer gives the probabilities of the surrounding segments. If two segments frequently appear in similar contexts in the training corpus, the weights of the hidden layer for these segments tend to become similar. At the end of the training, each segment is assigned a vector representation that corresponds to the weights of the hidden layer.

The whole-word model was trained using the Finnish internet corpus (Luotolahti et al., 2015) with unsegmented surface forms. Each subword model was trained separately using the same corpus, but prior to training, every word in the corpus was segmented according to the respective segmentation scheme. The word vector used in the subsequent decoding phase is the sum of the vectors corresponding to the segments that form the target word.

The dimension of the hidden layer and the context window size are controlled by hyperparameters. A context window size *N* indicates that *N* segments before and *N* segments after the target segment are considered in the training (Mikolov, Sutskever, et al., 2013, eq. 1). The size of the context window has been empirically shown to influence the degree to which vector representations emphasize syntactic versus semantic characteristics. For example, Bullinaria and Levy (2007) note that a reduced context window dimension yields optimal outcomes for a syntactic clustering task, while tasks with a semantic focus exhibit a performance trend that is comparatively less sensitive to variations in context window size. We trained all models with context window size 7 which has been shown to produce reasonable 300-dimensional word representations (Lapesa & Evert, 2014). We also examined the effect of context window size (from 2 to 7) on a subset of the models to determine the sensitivity of the approach to this parameter.

We additionally used hierarchical clustering of the word vectors of the different models to visualize the organization of the word vectors. We used the complete linkage algorithm, with cosine distance, which determines the distance between any clusters as the longest distance between any points in that cluster. The dendrograms for each model are included in the Supporting Information. As expected, in the whole-word, linguistic and Morfessor models, the organization of the word-vector space reflects a mixture of word meanings and morphological information. The character-level *n*-gram models reflect mostly character-based information, but when segmentation coincides with morphological suffixes, some morphological organization is evident. In random segmentation, the clustering is not readily interpretable.

## RESULTS

**Figure 4.**
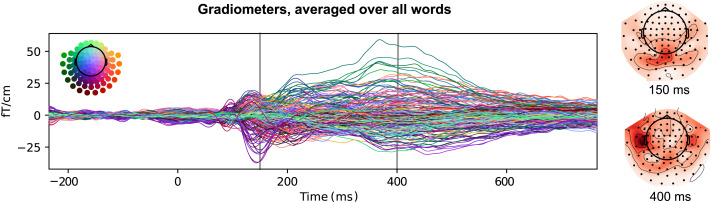
Time course of MEG signal amplitude. Signals recorded by the 204 MEG gradiometers are overlaid. Each word was first averaged across participants, and then an overall average was calculated across all words. Topographies are shown for the 150 ms and 400 ms time points, which are consistently associated with distinct neurofunctional responses in visual word recognition studies.

The sensor-level time courses of activation, averaged over the 170 words and 20 participants, are shown in Figure 4. The timing of peak amplitudes shows the typical pattern in a word reading task, from posterior transient responses within 200 ms after word onset to a sustained response in the temporal cortex between 200 and 600 ms.

**Figure 5.**
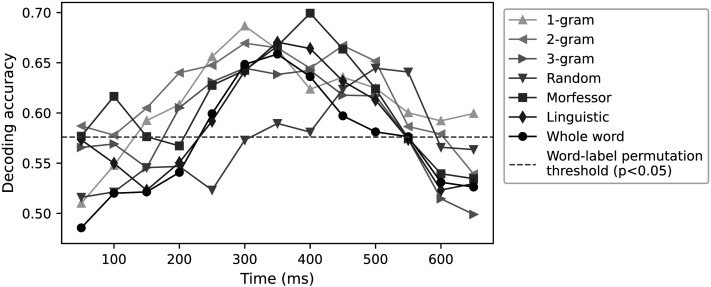
Decoding accuracy for corpus-semantic models as a function of time. The models are based on different subword units, and the models are visualized with different shades of gray and symbols. The dashed horizontal line is the significance threshold (*p* < 0.05), obtained through word-label permutation.

The results of the classification performance for each corpus-semantic model are shown in Figure 5 for context size 7. This figure illustrates the significance threshold obtained through word-label permutation, set at *p* < 0.05. The thresholds were calculated for each model separately; however, since they were similar across models, the highest value, 0.57, was adopted for use. All models reached significant results in the interval 350–500 ms. The decoding accuracy for the whole-word (black, circle), linguistic (diamond, dark gray), and Morfessor (square, gray) models showed relatively similar levels (0.65–0.69). The character-based 1-, 2- and 3-gram models reached comparable accuracies. The decoding accuracy of the random segmentation model (triangle down) remained slightly lower than that of the other models, with the maximum value at 0.64.

**Figure 6.**
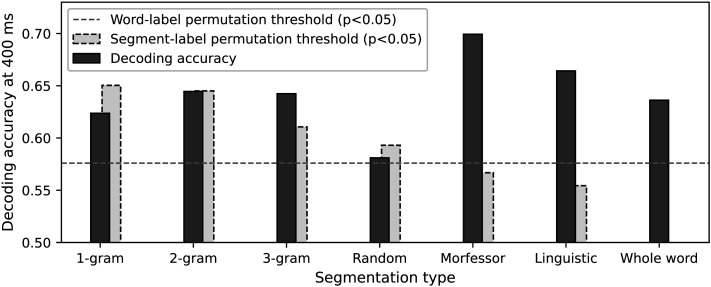
Decoding accuracy for corpus-semantic models at context size 7 for the best-performing time window centered around 400 ms (dark gray bar) and significance thresholds (*p* < 0.05) for segment-label permutation test (light gray bar), calculated for each model separately. The dashed horizontal line shows significance threshold (*p* < 0.05) of word-label permutation test (calculated for each model, highest value across all models shown). As the words in the whole-word model are not segmented, there is no associated segment-label permutation threshold.

Thus it seems all models are able to reach a reasonable decoding accuracy when compared to the chance level obtained by word-label permutation test. However, when we carried out the subword segment-label permutation, salient differences emerged between the models. In Figure 6, the decoding accuracy at 400 ms is compared to the chance level obtained by permuting the segment labels. The Morfessor and linguistic models yielded significant decoding accuracy. However, random segmentation no longer reached significance. Furthermore, for the character-based models, the subword chance levels markedly exceeded the word-label chance level, with the difference increasing systematically for smaller segments.

**Figure 7.**
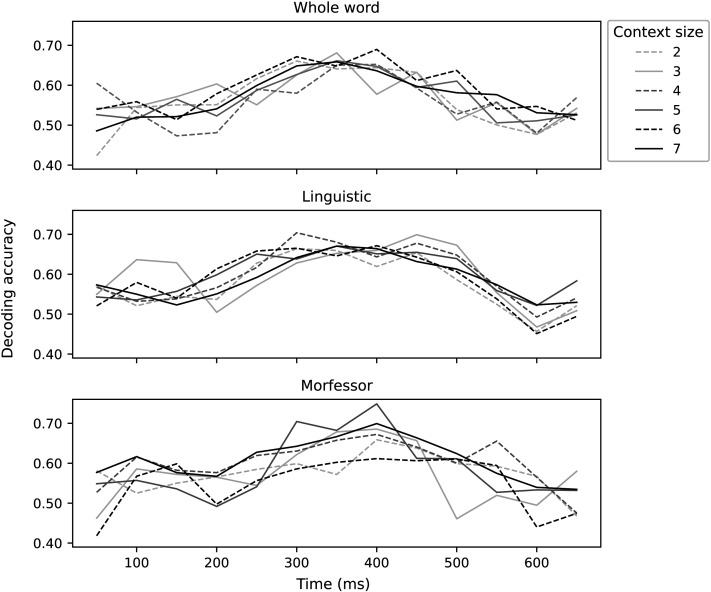
Decoding accuracy for context sizes 2–7 for the whole-word, linguistic, and Morfessor models. All context sizes show very similar decoding accuracy.

We evaluated the effect of the context size with a subset of the models. Figure 7 shows the effect of the context size for the whole-word, linguistic, and Morfessor models. The choice of this hyperparameter has little effect in the decoding results, justifying the use of the largest context size, 7, with all the models in the present study.

**Figure 8.**
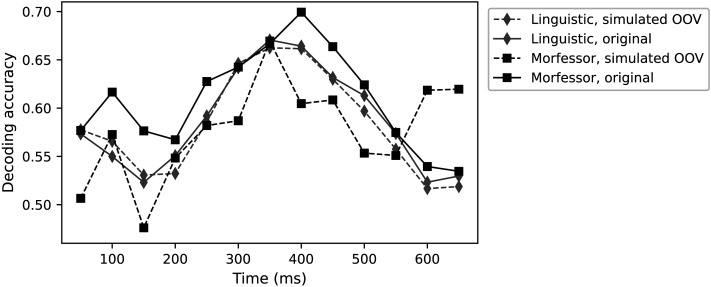
Decoding accuracy for the linguistic and Morfessor models when sentences containing the target words were excluded from the training corpus to simulate out-of-vocabulary (OOV) decoding. For comparison, results using the original corpus are also presented.

We additionally checked whether the decoding is possible only from subword segments that have never been a part of the original multimorphemic target word, thus, simulating performance for out-of-vocabulary items. We removed from the training corpus all the sentences that contained any of the multimorphemic 170 target words and repeated the experiment for Morfessor-derived and linguistic segmentations (Figure 8). The performance of the linguistic model remained almost unchanged when decoding out-of-vocabulary items. However, there was a decrease in performance for the Morfessor model. Notably, 27 words could not be represented using Morfessor segmentations, as there were no longer the required minimum of 50 instances of corresponding segments in the corpus to train reliable segment vectors. Consequently, they were omitted from the decoder training.

## DISCUSSION

We sought to determine whether cortical responses to multimorphemic words can be decoded using representations built as a sum of the vectors of their subword segments. We approached this question by recording MEG responses to multimorphemic words in a visual word recognition task, on the one hand, and building distributional corpus-semantic models of whole words, linguistic morphemes, statistical morphemes, and random word segments, on the other hand. Furthermore, to explore the limits of subword representations we additionally evaluated the performance of character-based 1-, 2- and 3-gram models. We linked these various models to the MEG measures using ridge regression. The success of this mapping, and thus the effect of the segmentation, was evaluated by predicting from the MEG data which word the participant was reading.

### Successful Decoding of Brain Responses to Words Using Subword Representations

The decoding accuracy reached around 0.65 using a corpus-semantic model of whole words which did not include additional information about morphology. Similar accuracy was achieved with the morpheme-based models which did not include the exact whole-word units. This level is on par with the results of previous studies that have used distributional corpus-semantic models to decode MEG responses evoked by noninflected simple written nouns (Derby et al., 2018; Hultén et al., 2021; Simanova et al., 2014; Sudre et al., 2012; Xu et al., 2016). The decoding was performed using sensor-level MEG data that were averaged over participants. Thus, although there is notable interindividual variation of spatiotemporal functional patterns, overall, the item-level MEG signals obtained by averaging across different participants nonetheless incorporated systematic between-item variation that enabled successful decoding.

The analysis of MEG data provided time-sensitive decoding accuracy. The accuracy exceeded significance threshold at 350–500 ms. This time window has been consistently associated with semantic and morphosyntactic processing using MEG (Fruchter & Marantz, 2015; Helenius et al., 1998; Service et al., 2007; Sudre et al., 2012; Vartiainen et al., 2009). A semantic effect around 400 ms that was dissociated from pre-lexical properties was also observed in intracranial electroencephalography (Hirshorn et al., 2016). In studies of morphological processing, the identification of morphemes has been associated with an earlier processing window at around 170 ms. (For a review of these findings, see, e.g., Leminen et al., 2018.) This processing stage has been, in most cases, linked to pre-lexical morphological decomposition or other processes that operate on the word-form level. Therefore, it seems probable that the decoding performance in the present study can be associated with semantic or syntactic properties rather than mere form-level features.

The decoding accuracies were remarkably similar for all models we studied. However, significance testing revealed that some of the models bore more relevance than others. As the goal was to examine summation of subword segments, it was essential to establish a significance threshold by permuting the segments, not merely the word labels, which is the typical approach. Both the segment-label and word-label chance levels highlighted corpus-based statistical (Morfessor) and linguistic subword models as well-functioning models of cortical activity evoked by words. However, for the corpus-based random segmentations and character-based 1- and 2-gram models, the decoding accuracy remained below the segment-label permutation threshold. For the 3-gram model, the decoding accuracy reached significance but even in that case the segment-label chance level notably exceeded the word-label chance level. This suggests that the individual subword vectors were not appropriate although, as a sum, they were able to decode the word label from the MEG signals.

### Decoding With Character-Based Models

We can try to understand the successful decoding using the character-based models in more detail. The boundaries defined by the segment-label permutation test, shown in Figure 6, can be loosely interpreted as a measure of the inherent structure within the set of word vectors. This structure emerges from the alignment of word vectors due to shared components, which reflects patterns of shared segments across words. The alignment facilitates effective decoding and functions independently of the specific information in each segment vector. In the extreme case of 1-gram model, the segments consist only of individual characters which are unlikely to carry meaningful semantic information. Since the positions of the characters are not considered, words that share the same characters are reduced to identical word vectors. Why would the evoked brain responses correspond to the common segments found in the words? Either the shared characters themselves or another correlated feature enable decoding. Given that all stimulus words are multimorphemic and have one or more regular suffixes, one possibility is that words sharing several common characters (or 2-grams) are, on average, more likely to contain identical suffixes. Consequently, words sharing grammatical categories tend to cluster together, at least to some extent. Visualization of word clustering using dendrograms (included in the Supporting Information), provides some support for this hypothesis as clusters of words with similar suffixes appear in the 1- and 2-gram dendrograms. However, drawing definite conclusions based on the present results is difficult.

As segment length extends to 3-grams, the segments become more individuated and some segments correspond to actual morphemes and words, enabling word2vec to endow these segments with more meaningful information. In the case of the random model, the threshold for segment-label permutation is lower compared to that of *n*-gram models, suggesting that there is less inherent structure due to shared segments compared to *n*-grams. Furthermore, the decoding accuracy also stays below this threshold, indicating that the individual segments are not informative enough for successful decoding. In the models that more closely approximate real morphemes, there are many long segments corresponding to word roots that are mostly unique within the stimulus set. The majority of the organization of the word-vector space is then a function of how word2vec organizes the segment vectors in relation to each other. Therefore, only the corpus-based Morfessor-derived and linguistic subword segments seem to contain semantic information such that their sum is comparable to the semantics of the whole word.

### Relevance to Morphological Processing in the Brain

The details of word segmentation in the human brain and morphological processing is an active area of neurolinguistics. There is still no clear consensus on the specifics of the processing despite the abundance of both data and theoretical accounts (Leminen et al., 2016; Leminen et al., 2018). For example, there are different views regarding how the brain learns which subword segments correspond to morphemes, and whether the meanings of the different morphemes are accessed separately before that of the whole word (i.e., the sublexical hypothesis; Taft, 1994) or whether morphological information is considered only after the whole word has been represented (i.e., the supralexical hypothesis in Giraudo & Grainger, 2001). Even the need for distinct morphemic representations linking orthography and semantics has been called into question (Baayen et al., 2011; Milin et al., 2017).

Our present results suggest that corpus-based statistical and linguistic segmentations both provided subword vectors that carried semantic relevance and that summation of those subword vectors served as an equally good model of brain-level word representations as a whole-word model. The summed subword vectors worked also when the original multimorphemic target words had been removed from the training corpus, thus the relevant information for decoding came from the other appearances of those subword segments in the training material. If we assume that the success of the model in predicting neural activity reflects some similarity between the representations described by the model and those present in the human brain, then our findings may be interpreted to suggest that morphemes are represented in the brain, along with the whole-word forms. To directly assess morphemic representations in the brain, one would need to show participants word segments, not complete multimorphemic words; however, such stimuli would seem quite strange to a human.

From the practical experimental point of view, the present method using subword representations provides a means of decoding multimorphemic words from brain data. Accordingly, the subword compositionality demonstrated here would enable experimenting also with words for which there is no statistically reliable surface representation in any large corpus, and even with pseudowords, as long as they are composed of word-like parts. This approach was here evaluated on the agglutinative Finnish language, and future studies are needed to examine its applicability to other types of languages.

### Limitations

In the current study, we utilize the Morfessor model as an example of a statistical approach where morphological information is derived in an unsupervised manner. Several other word models can leverage morphological regularity, but they are not tested in this study. For example, FastText (Bojanowski et al., 2017) encodes the word vector as a sum of all character *n*-grams of a word, and could also be used in the decoding tasks. Based on FastText word vectors, Nikolaev et al. (2022) constructed a generative model for multimorphemic Finnish words that represents word as a summation of latent vectors representing the meanings of its lexeme and its inflectional features. Even more elaborate representations (Marelli & Baroni, 2015), in which word suffixes are represented as matrices and a morphologically complex word is represented by multiplying a stem vector with a suffix matrix could be possible. Transformer-based architectures, which are capable of encoding token positions, also seem to be naturally suited for the task (Devlin et al., 2019).

The 2 versus 2 test is a common metric of classification accuracy. In the present study design, it assesses the ability to choose between two words with above chance accuracy, which may be viewed as a relatively weak notion of brain decoding. Nevertheless, it allows comparison between word segmentation models. Other applications for brain decoding might require ability to identify the word from a larger set of possibilities.

Beyond corpus-derived word vectors, it may be possible to enhance the classifier with extra information, like word frequencies and a range of other features. However, these aspects were not tested in this study, as the focus was on segmentation models. Distinguishing the influence of frequency from the already encoded semantic and contextual information in these vectors poses a considerable challenge.

The stimuli used were multimorphemic Finnish nouns, which is, naturally, only one word class. Whether the results can be generalized to other word classes, such as verbs, which may be processed differently in the brain remains to be explored.

## CONCLUSIONS

Our results suggest that while decoding accuracy of all models exceeded the typically used significance threshold for word-label permutation test, the critical segment-label permutation test revealed that only those segmentations that were morphologically aware reached significance in the brain decoding task. The observation that neural decoding of multimorphemic word forms can be achieved with corpus-semantic word representations derived from compositional subwords is especially relevant for study on languages with complex morphology where a large proportion of word forms are rare and it can be difficult to find statistically reliable surface representations for them in any large corpus. This study demonstrates that decoding is possible using purely information-theoretic principles, without a priori knowledge about the semantics or morphological structures of the language, thus mitigating the conceptual gap between linguistics and neuroscience. This opens avenues for more quantitative exploration of combinatorial processing mechanisms in the brain. These findings can inform the development of advanced language learning tools and more sophisticated computational models that better mimic the brain’s processing of language.

## ACKNOWLEDGMENTS

We would like to express our gratitude to Jenna Kanerva and Filip Ginter at TurkuNLP, University of Turku for collaboration on the development of the Finnish language word2vec models and Lingsoft Oy for the use of the linguistic analysis tool.

## FUNDING INFORMATION

Riitta Salmelin, Academy of Finland (https://dx.doi.org/10.13039/501100002341), Award ID: LASTU, 256887. Riitta Salmelin, Academy of Finland (https://dx.doi.org/10.13039/501100002341), Award ID: 255349. Riitta Salmelin, Academy of Finland (https://dx.doi.org/10.13039/501100002341), Award ID: 315553. Minna Lehtonen, Academy of Finland (https://dx.doi.org/10.13039/501100002341), Award ID: 288880. Annika Hultén, Academy of Finland (https://dx.doi.org/10.13039/501100002341), Award ID: 287474. Tiina Lindh-Knuutila, Aalto Brain Center. Riitta Salmelin, Sigrid Juséliuksen Säätiö (https://dx.doi.org/10.13039/501100006306). Riitta Salmelin, Academy of Finland, Award ID: 355407.

## AUTHOR CONTRIBUTIONS

**Tero Hakala**: Conceptualization; Data curation; Formal analysis; Investigation; Methodology; Software; Validation; Visualization; Writing – original draft; Writing – review & editing. **Tiina Lindh-Knuutila**: Conceptualization; Data curation; Formal analysis; Methodology; Sofware; Validation; Visualization; Writing – original draft; Writing – review & editing. **Annika Hultén**: Conceptualization; Investigation; Methodology; Software; Writing – original draft; Writing – review & editing. **Minna Lehtonen**: Conceptualization; Methodology; Writing – original draft; Writing – review & editing. **Riitta Salmelin**: Conceptualization; Funding acquisition; Methodology; Project administration; Resources; Supervision; Writing – original draft; Writing – review & editing.

## CODE AND DATA AVAILABILITY STATEMENTS

Ethics statement prevents sharing the raw research data. The data used to run the decoding experiments are publicly available at OSF: https://doi.org/10.17605/OSF.IO/2CBZW. These data include the MEG data averaged across participants, the word2vec vectors, and the experiment stimuli. The code is available at GitHub: https://github.com/AaltoImagingLanguage/Hakala2024/releases/tag/v1.0.0.

## Supplementary Material



## TECHNICAL TERMS

Corpus-semantic vector space: Captures semantic, syntactic, and functional relationships between word forms using statistical information from large text corpora.
Magnetoencephalography (MEG): A functional neuroimaging technique for measuring brain activity by recording magnetic fields produced by electrical currents that occur naturally in the brain.
Subword segment: A segment of characters that are part of a word; may or may not be a morpheme.
Brain decoding: Refers to predicting the stimulus word the participant is viewing from the measured MEG response to that word.
