# Supplementary material for: Subword Representations Successfully Decode Brain Responses to Morphologically Complex Written Words
Source: Neurobiol Lang (Camb). 2024 Sep 11;5(4):844–63. doi: 10.1162/nol_a_00149 (PMC11410357; doi:10.1162/nol_a_00149)
Supplement: Supplementary file 1 [file nol-5-4-844-s001.pdf]

Supplementary material 1. Target words and their segmentations

Table 1: The target words, their segmentations, English translation and the morphology of the target word. In the Morphology column, Case refers to the cases of Finnish nouns. Num refers to the singular (S) or plural (Pl) form. If Num value is missing, the word is ambiguous with regards to the number. Num[ps] refers to the singular or plural form of the possessive suffix, and Per[ps] to the person of the possessive suffix. Finnish 'nsa' third person possessive suffix is ambiguous with regards to the number. In the English translations, "their" thus refers to both singular and plural third person. When the number of the possessive suffix is not mentioned word is ambiguous in that regard. Finnish language also contains clitics (Cl) kin and kaan, which mean "also" and "neither", respectively, the third clitic "han" serves several different functions that make the meaning of a sentence more affirmative, infer shared knowledge or make an imperative more polite. Derivations (Der) "ja" and "minen" are used to derive nouns out of verbs in a similar way as in English: perform ⟶ performance or dance ⟶ dancing. The translation of the morphologically complex word into English is approximate. Depending on the context, other interpretations are possible. For example, the Finnish partitive form can be translated either as "some" or "part of". Below, both alternatives are used depending on the target word to provide the most common interpretation for a Finnish speaker. Finnish language does not have determiners, so they have been left out from the English translations.

**Whole word**	**Linguistic**	**Morfessor**	**Random**	**English**	**Morphology**
porukkamme	porukka mme	porukka mme	po r uk k amme	our team	Case=Gen Num=S Num[ps]=Pl Per[ps]=1
koreografian	koreografia n	koreografia n	k o r e ografian	choreography's	Case=Gen Num=S
saksofonistina	saksofonisti na	saksofonisti na	saksofo nis tina	as saxophonist	Case=Ess Num=S
pelkoakin	pelko a kin	pelkoa kin	pelkoa ki n	also fear	Case=Par Cl=Kin Num=S
naapurilta	naapuri lta	naapuri lta	naa pur ilt a	from neighbour	Case=Abl Num=S
valolle	valo lle	valo lle	v alolle	to light	Case=All Num=S
stressillä	stressi llä	stressi llä	s tre ss illä	with stress	Case=Ade Num=S
hidastaminen	hidasta minen	hidasta minen	hi das ta m in en	slowing down	Case=Nom Der=Minen Num=S
eläinteni	eläin t e ni	eläinten i	eläin te n i	my animals'	Case=Gen Num=Pl Num[ps]=S Per[ps]=1
monologissaan	monologi ssa an	monologi ssaan	m ono l o gissa an	in their monologue	Case=Ine Num=S Per[ps]=3
tekstien	tekst i en	tekstien	t eksti e n	texts'	Case=Gen Num=Pl
valsseista	valsse i sta	val sseista	valss eis ta	from waltzes	Case=Ela Num=Pl
otsikkokin	otsikko kin	otsikko kin	o tsikko kin	also headline	Case=Nom Cl=Kin Num=S
vipuun	vipu un	vi puun	vipuu n	into lever	Case=Ill Num=S
papereihinsa	papere i hi nsa	papereihin sa	papereih insa	into their papers	Case=Ill Num=Pl Per[ps]=3
miestensä	mies t e nsä	miesten sä	mi es t ensä	their mens'	Case=Gen Num=Pl Per[ps]=3
perustajille	perusta j i lle	perus tajille	per ustaj i ll e	founder	Case=All Num=Pl
sumuja	sumu j a	sumu ja	s umu ja	some mists	Case=Par Num=Pl
tieltäni	tie ltä ni	tieltä ni	tielt äni	from my road	Case=Abl Num=S Num[ps]=S Per[ps]=1
propagandassa	propaganda ssa	propaganda ssa	propaga ndas s a	in propaganda	Case=Ine Num=S
ohjaajana	ohjaa ja na	ohjaajana	o hjaaja na	as director	Case=Ess Num=S
höyheniään	höyhen i ä än	höyhen iään	höy he ni ää n	some of their feathers	Case=Par Num=Pl Per[ps]=3
tölkeissä	tölke i ssä	tölkeissä	tö lk ei ssä	in jars	Case=Ine Num=Pl

soittajalta	soitta ja lta	soittaja lta	so it ta jalt a	from player	Case=Abl Num=S
varoittaminen	varoitta minen	varoitta minen	varoittam inen	warning	Case=Nom Der=Minen Num=S
dokumentista	dokumenti sta	dokumentista	dokum e nti sta	from document	Case=Ela Num=S
sohvasi	sohva si	sohva si	sohva si	your sofa	Case=Nom Num=S Num[ps]=S Per[ps]=2
lisääntymisellä	lisäänty mise llä	lisääntymis ellä	l isä äntymis ellä	with reproduction	Case=Ade Der=Minen Num=S
johtamiseksi	johta mise ksi	johtamis eksi	johtami seksi	into leadership	Case=Tra Der=Minen Num=S
nuolia	nuol i a	nuoli a	nuo li a	some arrows	Case=Par Num=Pl
kanoottiin	kanootti in	kanootti in	kanoottii n	into canoe	Case=Ill Num=S
artikulaatiota	artikulaatio ta	artikulaatio ta	art ikul aa tiota	some articulation	Case=Par Num=S
maisemaan	maisema an	maisemaan	ma is e maan	into view	Case=Ill Num=S
pettymyksemme	pettymykse mme	pettymy ksemme	pet tymyksemme	our disappoint-ment	Case=Nom Num=S Num[ps]=Pl Per[ps]=1
kantamuksia	kantamuks i a	kanta muksia	kan tamuk sia	our burdens	Case=Par Num=Pl
näkemisenä	näke mise nä	näkemisen ä	näkemisen ä	as seeing	Case=Ess Der=Minen Num=S
psyykeen	psyyke en	psyyke en	psyyk een	into psyche	Case=Ill Num=S
pienuutensa	pienuute nsa	pien uutensa	pie nuute nsa	their smallness	Case=Gen Num=S Per[ps]=3
grafiikkaan	grafiikka an	grafiikka an	gra f iikkaa n	into graphics	Case=Ill Num=S
porojen	poro j en	poro jen	por ojen	reindeers'	Case=Gen Num=Pl
draamaa	draama a	draamaa	dr aa maa	some drama	Case=Par Num=S
taskustasi	tasku sta si	tasku stasi	t as kustas i	from your pocket	Case=Ela Num=S Num[ps]=S Per[ps]=2
mömmöjä	mömmö j ä	mömmö jä	m ömmöjä	some mucks	Case=Par Num=Pl
teknologioiden	teknologio i den	teknologioiden	te knol ogi o id e n	technologies'	Case=Gen Num=Pl
selaimesta	selaime sta	selaimesta	sel aimesta	from browser	Case=Ela Num=S
merkistöä	merkistö ä	merkistö ä	merk i st öä	part of character set	Case=Par Num=S
probleemasta	probleema sta	probleema sta	p r oble e ma sta	from a problem	Case=Ela Num=S
siskojen	sisko j en	siskojen	s isko je n	sisters'	Case=Gen Num=Pl
tulokkaisiin	tulokka i siin	tulo kkaisiin	t ul o k kai siin	into newcomers	Case=Ill Num=Pl
suunsa	suu nsa	suu nsa	s uun sa	their mouth	Case=Nom Num=S Per[ps]=3
kattilaan	kattila an	kattilaan	kattil aa n	into kettle	Case=Ill Num=S
hattusi	hattu si	hattu si	hattus i	your hat	Case=Nom Num=S Num[ps]=S Per[ps]=2
laskimossa	laskimo ssa	laskimo ssa	l a skimoss a	in vein	Case=Ine Num=S
lääkäreineen	lääkäre ine en	lääkäre ineen	lääkär ei n een	with their doctors	Case=Com Per[ps]=3
uhmalla	uhma lla	uhma lla	uh mal la	with defiance	Case=Ade Num=S
lakanat	lakana t	lakana t	laka na t	bedsheets	Case=Nom Num=Pl
kokoukseensa	kokouk see nsa	kokoukseen sa	kokou kseen sa	into their meeting	Case=Ill Num=S Per[ps]=3

varpaani	varpaa ni	varpaan i	varp a an i	my toe	Case=Gen Num=S Num[ps]=S Per[ps]=1
lehtineen	leht ine en	lehti neen	leh ti neen	with their leaf	Case=Com Per[ps]=3
viidakoiden	viidako i den	viida koiden	viid akoiden	jungles'	Case=Gen Num=Pl
klubilla	klubi lla	klubilla	k lu bil la	at club	Case=Ade Num=S
pesulat	pesula t	pesula t	p es ulat	laundries	Case=Nom Num=Pl
jumalakaan	jumala kaan	jumala kaan	j um alaka a n	even god	Case=Nom Cl=Kaan Num=S
kimpussani	kimpu ssa ni	kimpu ssani	ki mp ussan i	in my bouquet	Case=Ine Num=S Num[ps]=S Per[ps]=1
lihassani	liha ssa ni	liha ssani	lihas san i	in my meat	Case=Ine Num=S Num[ps]=S Per[ps]=1
shamanismiin	shamanismi in	shamanismi in	sh amani smii n	into shamanism	Case=Ill Num=S
mikseriä	mikseri ä	mikseri ä	mi ks eriä	part of mixer	Case=Par Num=S
lauluhan	laulu han	laulu han	lau luha n	song, isn't it	Case=Nom Cl=Han Num=S
kaarteesta	kaartee sta	kaarte esta	ka a rtee sta	from curve	Case=Ela Num=S
mediaa	media a	media a	m edia a	part of media	Case=Par Num=S
joukkueella	joukkuee lla	joukkueella	jo ukk ueel l a	with team	Case=Ade Num=S
instituutiot	instituutio t	instituutio t	in st itu utio t	institutions	Case=Nom Num=Pl
leikkaamossa	leikkaamo ssa	leikkaamo ssa	l ei kk aa mossa	in cutting room	Case=Ine Num=S
lapsikaan	lapsi kaan	lapsi kaan	laps i kaa n	even (a) child	Case=Nom Cl=Kaan Num=S
algoritmeja	algoritme j a	algoritmeja	algoritme ja	some algorithms	Case=Par Num=Pl
pakkaseen	pakka seen	pakkas een	pakk asee n	into frost	Case=Ill Num=S
ruhtinaan	ruhtinaa n	ruhtinaan	r uhtinaa n	duke's	Case=Gen Num=S
taidoissa	taido i ssa	taidoissa	t aidois s a	in skills	Case=Ine Num=Pl
päärynällä	päärynä llä	päärynä llä	päär ynällä	with pear	Case=Ade Num=S
symboleilla	symbole i lla	symboleilla	sym bo leil l a	with symbols	Case=Ade Num=Pl
poliitikoilta	poliitiko i lta	poliiti koilta	pol iitikoilta	from politicians	Case=Abl Num=Pl
yllyttämiseksi	yllyttä mise ksi	ylly ttämiseksi	yl lyttämisek si	into incitement	Case=Tra Der=Minen Num=S
keilailussa	keilailu ssa	keilailu ssa	keilai lussa	in bowling	Case=Ine Num=S
animaatiota	animaatio ta	animaatio ta	an ima atiota	part of animation	Case=Par Num=S
sykliä	sykli ä	sykli ä	sykl iä	part of cycle	Case=Par Num=S
järjestöjen	järjestö j en	järjestöjen	jä r je stöjen	organizations'	Case=Gen Num=Pl
ystävällenne	ystävä lle nne	ystävälle nne	y stävällen ne	for your friend	Case=All Num=S Num[ps]=Pl Per[ps]=2
potilaita	potila i ta	potilaita	pot ilait a	some patients	Case=Par Num=Pl
mullaksi	mulla ksi	mulla ksi	mul l aks i	into soil	Case=Tra Num=S
kyvyistä	kyvy i stä	kyvyistä	ky vyistä	from abilities	Case=Ela Num=Pl
haukkumisia	haukku mis i a	haukku misia	ha ukk um is ia	some barkings	Case=Par Der=Minen Num=Pl
kuvaamisensa	kuvaa mise nsa	kuvaa misensa	k u vaamisensa	their photography	Case=Gen Der=Minen Num=S Per[ps]=3
velkaantumisena	velkaantu mise na	velkaantumis ena	ve lk aant umi s e na	as indebtness	Case=Ess Der=Minen Num=S

parvekkeeksi	parvekkee ksi	parve kkeeksi	parvekke ek s i	into balcony	Case=Tra Num=S
kontaktein	kontakte in	kontakt ein	with kontakt ein	contact	Case=Ins Num=Pl
pyssyjen	pyssy j en	pyssyjen	pyssyj en	guns’	Case=Gen Num=Pl
haitarin	haitari n	haitari n	h aitarin	accordion’s	Case=Gen Num=S
teoriasi	teoria si	teoria si	te o rias i	your theory	Case=Nom Num=S Num[ps]=S Per[ps]=2
selitteitä	selitte i tä	selit teitä	sel i tteitä	some legends	Case=Par Num=Pl
kollegoiltani	kollego i lta ni	kollego ilta ni	k ol l egoi ltan i	from my colleagues	Case=Abl Num=Pl Num[ps]=S Per[ps]=1
kaasuakin	kaasu a kin	kaasua kin	ka a suaki n	also some gas	Case=Par Cl=Kin Num=S
kaarella	kaare lla	kaarella	ka ar ell a	on a bow	Case=Ade Num=S
maineessa	mainee ssa	mainee ssa	ma ineess a	in reputation	Case=Ine Num=S
koomikkoja	koomikko j a	koomikko ja	koomi kkoja	some comedians	Case=Par Num=Pl
esiintymisestä	esiinty mise stä	esiintymis estä	e siin ty mi s estä	from performance	Case=Ela Der=Minen Num=S
loiset	loise t	loiset	l oiset	parasites	Case=Nom Num=Pl
pyörteet	pyörtee t	pyör teet	pyörtee t	vortices	Case=Nom Num=Pl
lajikkeet	lajikkee t	lajikkeet	laj ikkee t	cultivars	Case=Nom Num=Pl
äitiyttä	äitiy ttä	äiti yttä	äit iytt ä	part of maternity	Case=Par Num=S
hermojani	hermo j a ni	hermoja ni	h ermoja ni	some of my nerves	Case=Par Num=Pl Num[ps]=S Per[ps]=1
sikiöstä	sikiö stä	sikiö stä	sikiös tä	from fetus	Case=Ela Num=S
arvionaan	arvio na an	arvion aan	arvio na an	as their estimate	Case=Ess Num=S Per[ps]=3
varaamiseen	varaa mise en	varaa miseen	va raam is e en	to reserving	Case=Ill Der=Minen Num=S
talvikaan	talvi kaan	talvi kaan	talvi kaan	even winter	Case=Nom Cl=Kaan Num=S
ajatusta	ajatus ta	ajatusta	a jatusta	part of thought	Case=Par Num=S
vaimolta	vaimo lta	vaimo lta	vai molta	from wife	Case=Abl Num=S
tutustuminenkin	tutustu minen kin	tutustu minenkin	tut ustumin en kin	also meeting	Case=Nom Cl=Kin Der=Minen Num=S
singlestä	single stä	single stä	s inglestä	from single	Case=Ela Num=S
tikkarin	tikkari n	tikkari n	tikkar in	lollipop’s	Case=Gen Num=S
frendejä	frende j ä	fren dejä	fr en d ejä	some friends	Case=Par Num=Pl
asiakkaitamme	asiakka i ta mme	asiakkaitamme	asi akkai t amme	some of our customers	Case=Par Num=Pl Num[ps]=Pl Per[ps]=1
moskeijoissa	moskeijo i ssa	moskei joissa	mos keij o is sa	in mosques	Case=Ine Num=Pl
autismista	autismi sta	autismi sta	autis mista	from autism	Case=Ela Num=S
hoitoon	hoito on	hoitoon	h oito on	into treatment	Case=Ill Num=S
musikaaliksi	musikaali ksi	musikaali ksi	m usi k aa liks i	into musical	Case=Tra Num=S
veljenään	velje nä än	veljen ään	velj e nä än	as their brother	Case=Ess Num=S Per[ps]=3
pakaroita	pakaro i ta	pakaro ita	pa ka r oita	some buttocks	Case=Par Num=Pl
hippuset	hippuse t	hippu set	h ippuse t	pinches	Case=Nom Num=Pl
kengässä	kengä ssä	kengässä	ke ngä s sä	in shoe	Case=Ine Num=S
injektion	injektio n	injektio n	in j ekti on	injection’s	Case=Gen Num=S

pakkaamoon	pakkaamo on	pakkaamo on	pakk aam oon	into packing room	Case=Ill Num=S
aurinkosi	aurinko si	aurinko si	au ri nko si	your sun	Case=Nom Num=S Num[ps]=S Per[ps]=2
löydöltä	löydö ltä	löydö ltä	löy döl tä	from finding	Case=Abl Num=S
projektistasi	projekti sta si	projektista si	pr ojektistasi	from your project	Case=Ela Num=S Num[ps]=S Per[ps]=2
rahdit	rahdi t	rahdit	r a hdit	freights	Case=Nom Num=Pl
jakkujen	jakku j en	jakku jen	j akk u jen	jackets'	Case=Gen Num=Pl
editoimiseen	editoi mise en	editoi miseen	edi t o im ise en	into editing	Case=Ill Der=Minen Num=S
jännäriksi	jännäri ksi	jännäri ksi	jännär i ks i	into thriller	Case=Tra Num=S
kahvimme	kahvi mme	kahvi mme	kahvi mm e	our coffee	Case=Gen Num=S Num[ps]=Pl Per[ps]=1
kypsymiselle	kypsy mise lle	kypsy miselle	kypsy m i s el le	for maturing	Case=All Der=Minen Num=S
uneksijan	uneksi ja n	uneksi jan	une ks ijan	dreamer's	Case=Gen Num=S
repussaan	repu ssa an	repussa an	r e pussaa n	in their backpack	Case=Ine Num=S Per[ps]=3
salakuljettajat	salakuljettaja t	sala kuljettajat	sal a kuljetta ja t	smugglers	Case=Nom Der=Ja Num=Pl
menetyksesi	menetykse si	menet yksesi	me ne tyk s esi	your loss	Case=Nom Num=S Num[ps]=S Per[ps]=2
faktaan	fakta an	fakta an	fa ktaan	into fact	Case=Ill Num=S
kuulonsa	kuulo nsa	kuulo nsa	k uulo n sa	their hearing	Case=Gen Num=S Per[ps]=3
sisaruksillaan	sisaruks i lla an	sisar uksilla an	s i saru ksi l la an	with their siblings	Case=Ade Num=Pl Per[ps]=3
indekseihin	indekse i hin	indeks eihin	ind e kse i hin	indices	Case=Ill Num=Pl
lepäämisen	lepää mise n	lepää misen	le pää m i sen	of resting	Case=Gen Der=Minen Num=S
palveluksiin	palveluks i in	palvelu ksiin	palveluk siin	into services	Case=Ill Num=Pl
asioistasi	asio i sta si	asioista si	as io istasi	your affairs	Case=Ela Num=Pl Num[ps]=S Per[ps]=2
linkkejä	linkke j ä	linkkejä	link kejä	some links	Case=Par Num=Pl
kampanjassaan	kampanja ssa an	kampanjassa an	kampan jassaan	in their campaign	Case=Ine Num=S Per[ps]=3
huoltamolta	huoltamo lta	huoltamo lta	h uoltam o lta	from gas station	Case=Abl Num=S
pistoolillaan	pistooli lla an	pistoolilla an	pist ooli lla a n	with their pistol	Case=Ade Num=S Per[ps]=3
luennoitsijalle	luennoitsi ja lle	luennoitsija lle	luenn oits ij al le	to lecturer	Case=All Num=S
tehojakin	teho j a kin	tehoja kin	teh ojaki n	also some power	Case=Gen Num=S
näytöllään	näytö llä än	näytöllä än	nä y t öllään	in their monitor	Case=Ade Num=S Per[ps]=3

ääntämme	ään tä mme	ääntä mme	ään täm me	some of our voice	Case=Par Num=S Num[ps]=Pl Per[ps]=1
naamoille	naamo i lle	naamo ille	na am oil le	to faces	Case=All Num=Pl
saalistajiin	saalista j i in	saalis tajiin	sa alist aj i in	into predator	Case=Ill Num=Pl
edustalle	edusta lle	edusta lle	e dus talle	to front	Case=All Num=S
hirvien	hirv i en	hirvi en	hi r vien	mooses'	Case=Gen Num=Pl
aistimuksia	aistimuks i a	aistimuksia	a i stim uksi a	some sensations	Case=Par Num=Pl
tilanteessasi	tilantee ssa si	tilanteessa si	t i l antee ss asi	in your situation	Case=Ine Num=S Num[ps]=S Per[ps]=2
selviytymisellä	selviyty mise llä	selviytymis ellä	selv i ytymisellä	with survival	Case=Ade Der=Minen Num=S
marjat	marja t	marjat	ma rja t	berries	Case=Nom Num=Pl
rahalta	raha lta	raha lta	r ahalta	for money	Case=Abl Num=S
puuhani	puuha ni	puuha ni	puu hani	my chore	Case=Nom Num=S Num[ps]=S Per[ps]=1
itämisessä	itä mise ssä	i tämisessä	itä mise s s ä	in germination	Case=Ine Der=Minen Num=S

Supplementary material 2: Hierarchical clustering of the corpus-derived vectors for different word segmentation models

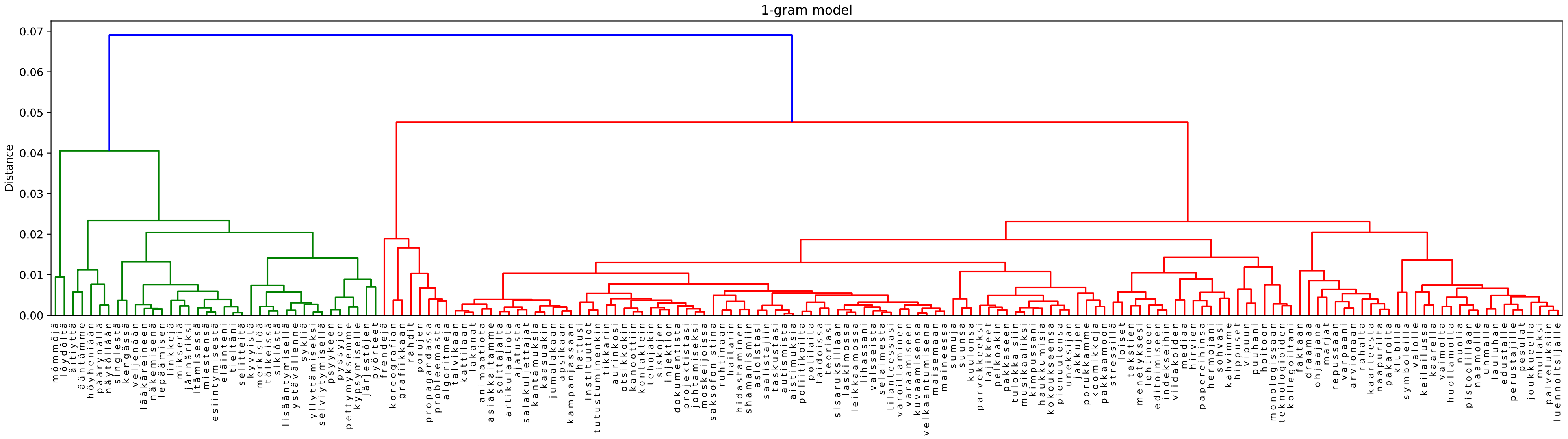

Figure 1: Hierarchical clustering of the corpus-derived vectors of the 1-gram model using cosine similarity.Distances between vectors are very small compared to other models. Organization of the vector space seems to be based on shared characters regardless of their order.

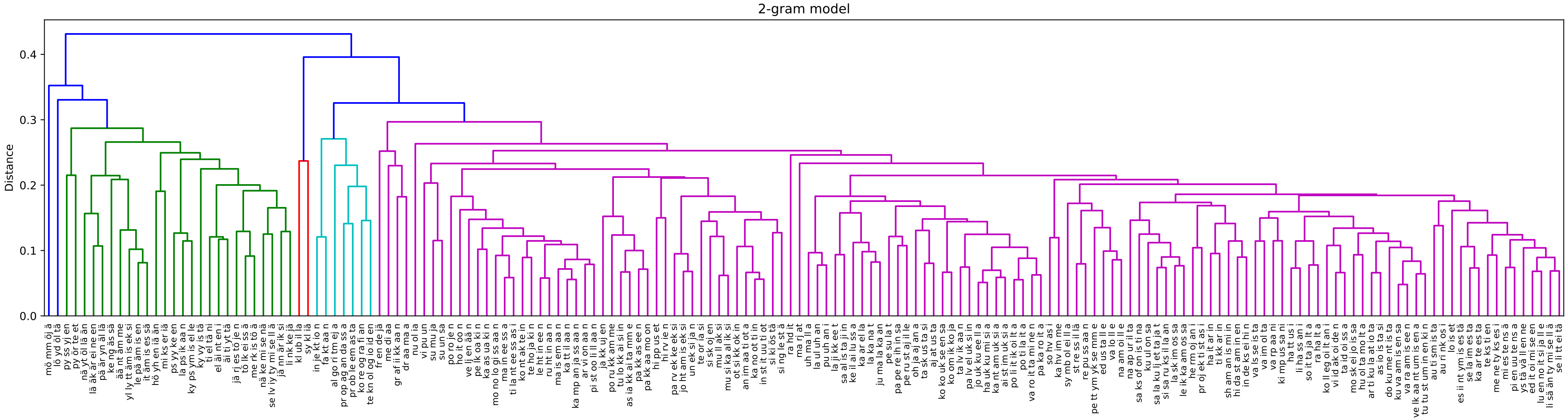

Figure 2: Hierarchical clustering of the corpus-derived vectors of the 2-gram model using cosine similarity. Organization is based on shared character-level n-grams. If shared character n-grams occur at the end of the word, they resemble morphological suffices, for example the partitive "ta", or genitive "n", but they can also occur anywhere, for example "la ka na t" (bedsheets) and "ju ma la ka an" (even god) are close to each other in the space.

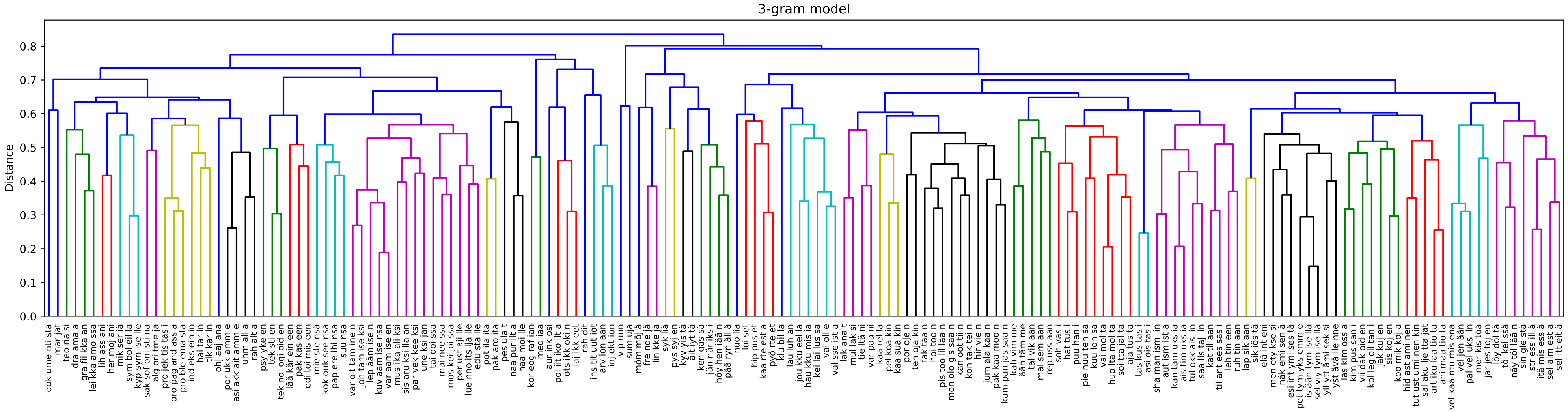

Figure 3: Hierarchical clustering of the corpus-derived vectors of the 3-gram model using cosine similarity. Organization of the space is based on shared character-level n-grams, but it resembles more an organization based on morphological suffices, as many suffices contain 3 characters, such as 1st person plural possessive suffix "mme", 3rd person possessive suffix "nsa", inessive case "ssa", elative case "sta", adessive "lla" and ablative "lta". No semantic organization is seen.

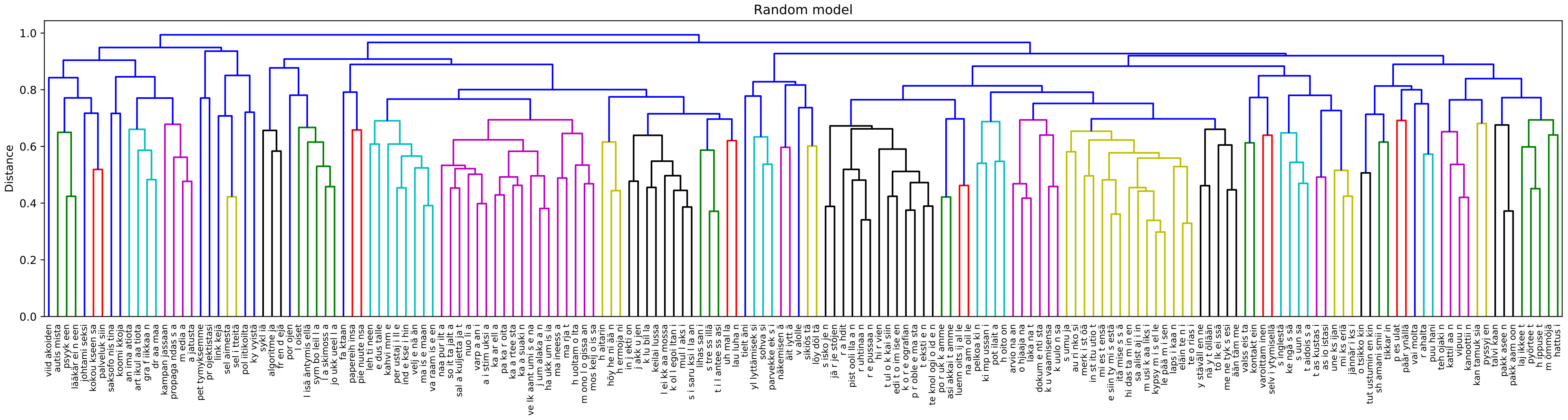

Figure 4: Hierarchical clustering of the corpus-derived vectors of the random segmentation model using cosine similarity. The organization is based on shared segments. Often a small segment such as "a" seems to derive the organization. There does not seem to be any organization based on semantics.

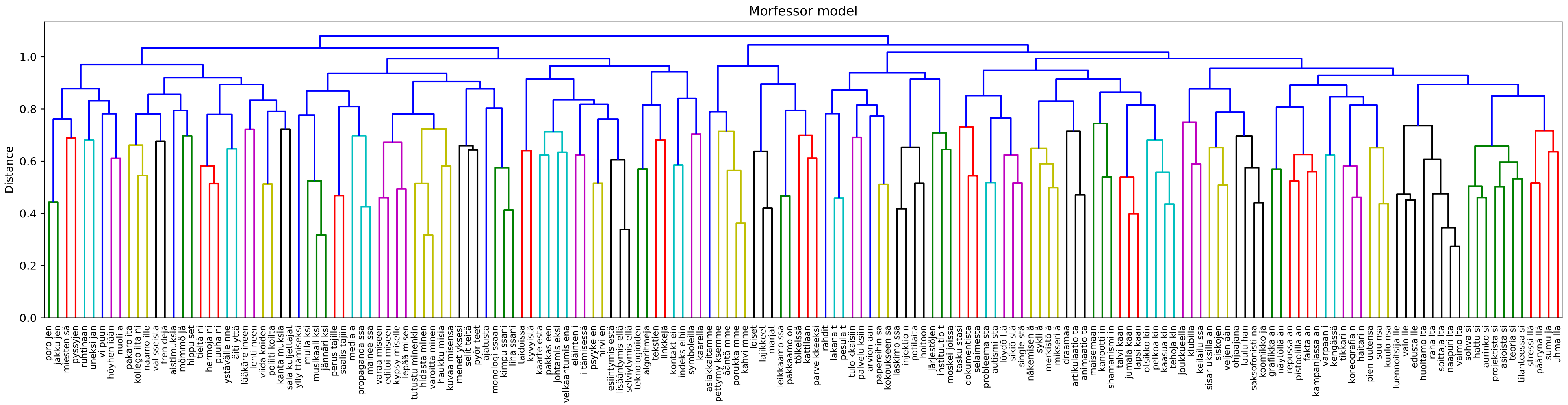

Figure 5: Hierarchical clustering of the corpus-derived vectors of the Morfessor model using cosine similarity. The organization seems to be a mix of semantic and morphological/suffix information with emphasis on the morphology. Some semantic categories such as the family relations "sisar uksilla an" (their siblings), "siskojen" (sisters'), and "veljen ään" (as brother) can be found.

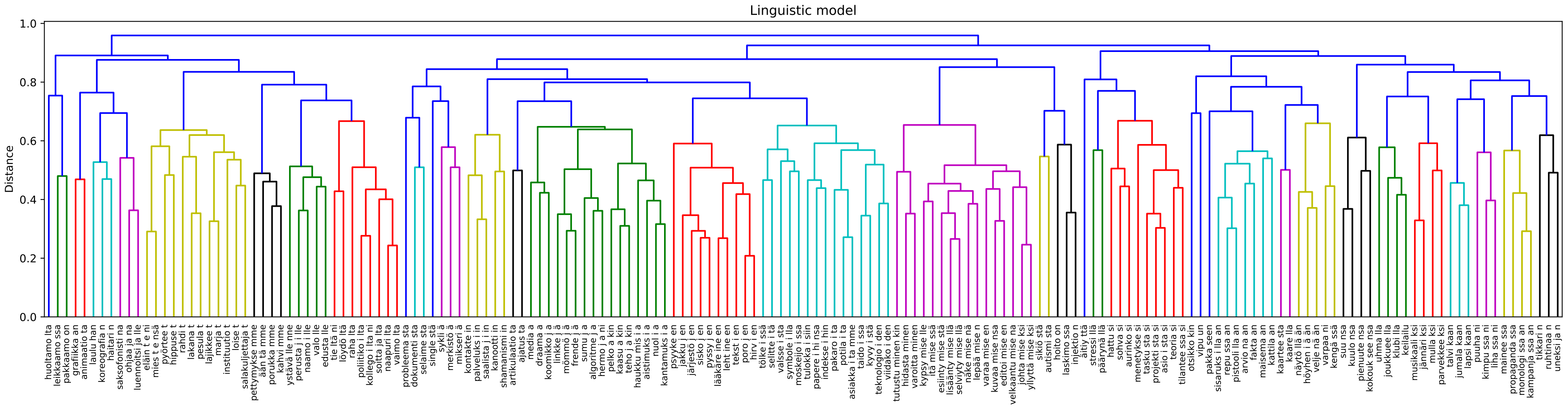

Figure 6: Hierarchical clustering of the corpus-derived vectors of the linguistic model using cosine similarity. The organization of the word-vector space seems to be a mix of semantic and morphological information: Higher-level clustering is based on morphology, but semantically meaningful pairs can be found within the clusters. For example in the nominative plural cluster (suffix "-t"), "lakana t" (bedsheets) and "pesula t" (laundries) form a pair, and "marja t" (berries) and "lajikkee t" (varieties) form another pair.

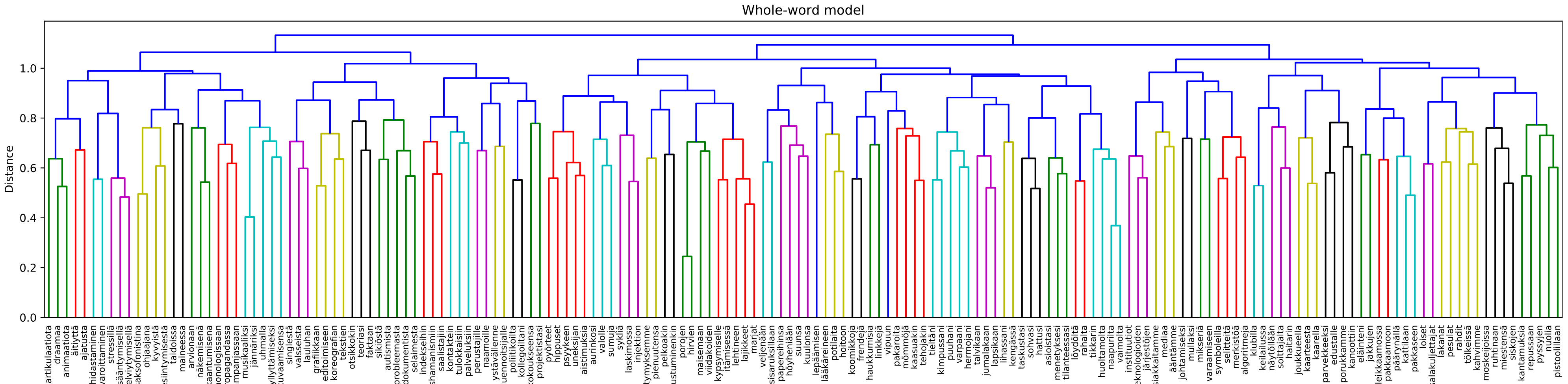

Figure 7: Hierarchical clustering of the corpus-derived vectors of the whole-word model using cosine similarity. The organization of the word-vector space seems to reflect the meanings, but there is also morphological organization, highlighting the fact that morphology also carries a meaning. For example "kin" (also) and first person possessive suffix "ni" form clusters.
